# Association of DNA Methylation with Infant Birth Weight in Women with Gestational Diabetes

**DOI:** 10.3390/metabo14070361

**Published:** 2024-06-27

**Authors:** Renata Saucedo, Aldo Ferreira-Hermosillo, Magalhi Robledo-Clemente, Mary Flor Díaz-Velázquez, Jorge Valencia-Ortega

**Affiliations:** 1 Unidad de Investigación Médica en Enfermedades Endocrinas, Hospital de Especialidades, Centro Médico Nacional Siglo XXI, Instituto Mexicano del Seguro Social, Mexico City 06720, Mexico; renata.saucedo@imss.gob.mx (R.S.); aldo.ferreira@imss.gob.mx (A.F.-H.); 2 Hospital de Gineco Obstetricia 3, Centro Médico Nacional La Raza, Instituto Mexicano del Seguro Social, Mexico City 02990, Mexico; ginemagalhi@outlook.es (M.R.-C.); mary.diaz@imss.gob.mx (M.F.D.-V.); 3 Unidad de Investigación en Reproducción Humana, Instituto Nacional de Perinatología-Facultad de Química, Universidad Nacional Autónoma de México, Mexico City 11000, Mexico

**Keywords:** DNA methylation, epigenetics, gestational diabetes, macrosomia, birth weight

## Abstract

Offspring exposed to gestational diabetes mellitus (GDM) exhibit greater adiposity at birth. This early-life phenotype may increase offspring risk of developing obesity, metabolic syndrome, type 2 diabetes, and cardiovascular disease later in life. Infants born to women with GDM have a dysregulation of several hormones, cytokines, and growth factors related to fetal fat mass growth. One of the molecular mechanisms of GDM influencing these factors is epigenetic alterations, such as DNA methylation (DNAm). This review will examine the role of DNAm as a potential biomarker for monitoring fetal growth during pregnancy in women with GDM. This information is relevant since it may provide useful new biomarkers for the diagnosis, prognosis, and treatment of fetal growth and its later-life health consequences.

## 1. Introduction

Gestational diabetes mellitus (GDM), which is defined as glucose intolerance with onset or first recognition in pregnancy and normalized after parturition, is the most common pregnancy complication [1]. According to the International Diabetes Federation, the global prevalence of GDM was 16.7% in 2021, and its incidence is rising, parallel to the growing prevalence of obesity and increasing maternal age [2]. Additional factors that increase the risk of GDM include increasing parity, family history of type 2 diabetes mellitus (T2DM), and past delivery of a macrosomic infant [3].

As pregnancy evolves, insulin resistance is increased in order to supply nutrients to the fetus. As a result, β-cell function must adapt in order to avoid the development of hyperglycemia. The most prominent features of GDM are insulin resistance and β-cell dysfunction. β-cell function will decrease by 30–70% in GDM, and β-cells cannot cope with the increased insulin resistance [4]. GDM is usually diagnosed between 24 and 28 weeks of gestation and there are various tests that can be applied. The most common is a one-step test using a 75 g, 2 h oral glucose tolerance test (OGTT) [1].

GDM is an important cause of pregnancy complications, such as premature rupture of membranes, pre-eclampsia, and gestational hypertension [5]. GDM may also cause fetal complications, including macrosomia, shoulder dystocia, cesarean section, premature birth, respiratory distress syndrome, hyperbilirubinemia, and neonatal hypoglycemia [6]. Moreover, GDM increases the risk of later development of metabolic syndrome, T2DM, and cardiovascular disease in mothers and their offspring [7,8,9]. Additionally, the risk of premature death is higher for women with GDM [10].

In pregnancies complicated by GDM, the newborns have shown an increased risk of altered growth patterns, including increased neonatal body fat and a higher birth weight [11]. Macrosomia, defined by excess body fat, is the most common fetal complication of GDM. Its prevalence in pregnancies complicated by maternal diabetes is 50% [12]. Macrosomia is defined as birth weight in full-term infants over 4000 g or when weight, at any gestational age, after adjustment for gender and ethnicity, surpasses the 90th percentile [13]. Macrosomia has life-long health implications for the infant, including an increased risk of developing obesity, chronic disease and some cancers [14]. Uncontrolled maternal hyperglycemia, hyperlipidemia, and obesity are factors known to be involved in the development of macrosomia [12,15]. However, accurately predicting macrosomia is still a challenge and the underlying molecular mechanisms are still poorly understood. There is growing evidence that epigenetic changes which regulate gene expression without a change in the nucleotide sequence contribute to the increased fetal growth [16]. Epigenetic mechanisms primarily include DNA methylation (DNAm), noncoding RNA, histone modifications, and chromatin remodeling. Growing evidence indicates that DNAm both in maternal and fetal tissues is involved in fetal growth [17]. The present narrative review will assess recent evidence on the role of DNAm as a potential biomarker for monitoring fetal growth during pregnancy in women with GDM. This knowledge will help to improve the early identification of macrosomia and will guide the future development of therapeutic strategies against this fetal complication.

Studies were identified between December of 2023 and May of 2024 by searching through the PubMed, Scopus, and Web of Science databases for papers that investigated the influence of DNAm in the placenta, umbilical cord blood (CB), maternal adipose tissue (AT), and maternal blood (MB) on the growth of fetuses exposed to intrauterine hyperglycemia. The search terms included the following: gestational diabetes mellitus, DNA methylation, macrosomia, epigenetics, and birth weight. This review included case/control studies that focused on DNAm in GDM women in association with fetal growth that were conducted in humans and were written in English.

## 2. General Aspects of DNAm

The most investigated epigenetic modification is DNAm, which consists of adding a methyl group (-CH3) to the five positions of cytosine residues, often located in a CpG dinucleotide in the promoter and exonic regions, leading to 5-methylcytosine formation. Three DNA methyltransferases (DNMTs), DNMT1, DNMT3a, and DNMT3b, participate in CpG dinucleotide methylation, using S-adenosylmethionine as the donor of methyl groups. This epigenetic mechanism is heritable, reversible, and can be induced by environmental factors [18].

CpG methylation is thought to include a range of 70–80% in DNA sequencing in human cells. The methylation of CpG dinucleotides typically leads to gene silencing while increasing the possibility of binding other proteins to the methylated promoter region, leading to the blocking of the transcription of the specific gene. Therefore, DNAm inhibits gene expression in a cell-type-specific manner [19]. However, few studies have shown a positive correlation between DNAm and gene expression [20].

Interestingly, DNAm is involved in several developmental processes like embryogenesis, genomic imprinting, X-chromosome inactivation, and multiple human diseases like cancer, obesity, T2DM, and cardiovascular, neurodegenerative, and neuropsychiatric disorders [21,22].

There are different techniques currently available to study DNAm. The first method is global methylation, which quantifies overall genomic methylation. The second method is the genome-wide approach, performing methylation arrays, and the third method is the gene-specific approach [23,24,25,26].

## 3. DNAm in GDM

The studies included in this review reported DNAm dysregulation in the placenta, CB, AT, and MB exposed to GDM [27,28,29,30,31,32,33,34,35,36,37,38,39,40,41,42,43,44,45]. It has been reported that maternal glucose levels at fasting, 1 and 2 h post-glucose load from the 24th to 28th week of gestation, fasting glucose at delivery, maternal insulin, lipid-derived metabolites, obesity, and oxidative stress influence the patterns of DNAm [46,47,48,49,50]. Nevertheless, it has been shown that other factors are involved. Research carried out by Gagné et al. showed that GDM exposure explained 7–14% of DNAm variability in the placenta and suggested that the remaining DNAm variability includes several prenatal and genetic factors [35].

Additionally, the biological relevance of the differentially methylated genes in the genome-wide approach studies included in this review was determined using Ingenuity Pathway Analyses (IPAs). Ruchat et al. showed that the principal pathways emerging from the placenta were cardiovascular diseases, metabolic diseases, and psychological disorders. In CB, the top pathways identified were gastro-intestinal diseases and metabolic and endocrine system disorders. The differentially methylated genes common to both tissues were related to immunological diseases, metabolic diseases, and endocrine system disorders [27]. On the other hand, the Kyoto Encyclopedia of Genes and Genomes (KEGG) pathway analysis performed by Yan et al. revealed enrichment for metabolic pathways, pathways in cancer, and an alteration in the insulin-signaling pathway in methylated genes [41]. Lastly, IPA analysis performed by Wang et al. identified that differentially methylated genes were most enriched in bladder cancer signaling, followed by GABA receptor signaling and uracil degradation. It should be noted that no pathway reached statistical significance when correcting for multiple tests [43].

### 3.1. Potential Clinical Applications of DNAm in GDM

Since differentially methylated genes have been identified in women with GDM, it has been suggested that this epigenetic modification may play a role in the pathogenesis of this hyperglycemic condition. To our knowledge, three studies have investigated DNAm profiles in maternal blood prior to GDM development. In a cohort, Wu et al. studied pregnant women between 12 and 16 weeks’ gestation and identified differential methylation in five CpGs within the constitutive photomorphogenic homolog subunit 8 (*COPS8*), phosphoinositide-3-kinase, regulatory subunit 5 (*PIK3R5*), 3-hydroxyanthranilate 3,4-dioxygenase (*HAAO*), coiled-coil domain containing 124 *(CCDC124*), and chromosome 5 open reading frame 34 (*C5orf34*) genes in women who developed GDM relative to matched healthy pregnancies. The authors performed a genome-wide analysis of DNAm using the Illumina HumanMethylation450 BeadChip, in addition to which they validated the data using pyrosequencing. The functions of differentially methylated genes are the following: *COPS8* regulates multiple signaling pathways, *PIK3R5* influences cell growth, proliferation, differentiation, motility, survival, and intracellular trafficking, and *HAAO* catalyzes the synthesis of quinolinic acid, which may participate in the pathogenesis of neurologic and inflammatory disorders. *CCDC124* enables RNA-binding activity and is involved in cell division, and finally, *C5orf34* has an unknown function and is involved in Diamond–Blackfan anemia 4 [51].

Similarly, Enquobahrie et al. investigated pregnant women at 16 weeks’ gestation using genome-wide DNAm analysis and found 17 hypomethylated and 10 hypermethylated CpG sites in the GDM group. The hypomethylated genes were hyaluronan and proteoglycan link protein 3 (*HAPLN3*); HERV-H LTR-associating 3 (*HHLA3*); ras homolog family member G (*RHOG*); MDM2 oncogene, E3 ubiquitin protein ligase (*MDM2*); DnaJ (Hsp40) homolog, subfamily B, member 6 (*DNAJB6*); interleukin 7 (*IL7*); yes-associated protein 1 (*YAP1*); cyclin-dependent kinase inhibitor 2B (*CDKN2B*); N-Acetylgalactosaminidase, α-(*NAGA*); chromosome 1 open reading frame 43 (*C1orf43*); NADH dehydrogenase (ubiquinone) I, subcomplex unknown (*NDUFC1*); NADH dehydrogenase (ubiquinone) 1 alpha subcomplex, 12 (*NDUFA12*); transmembrane and coiled-coil domains 4 (*TMCO4*); deleted in colorectal carcinoma (*DCC*); zinc finger protein 177 (*ZNF177*); variable charge, X-linked (*VCX*); and phosphodiesterase 6H, cGMP-specific, cone, γ (*PDE6H*); and the hypermethylated genes were Agouti-related protein (*AGRP*); proline rich, 22 (*MGC24975*); chromosome 2 open reading frame 27B (*MGC50273*); prostate and breast cancer overexpressed 1 (*PBOVI*); long intergenic nonprotein coding RNA 242 (*C6orf122*); tight junction-associated protein 1 (*TJAPI*); zinc finger, MYM-type 3 (*ZMYM3*); zygote arrest 1 (*ZARI*); discoidin domain receptor tyrosine kinase 1 (*DDRI*); and septin 11 (*SEPTII*). These genes participate in cell morphology, cellular assembly, cellular organization, cellular compromise, and cell cycle [52].

On the other hand, in a nested case/control study performed by Wang et al. in women during early pregnancy, methylation levels at six CpGs sites within the RHO Guanosine triphosphatase activating protein 40 (*ARHGAP40*), signal transducer and activator of transcription 1 (*STAT1*), *C5orf34*, retinol dehydrogenase 12 (*RDH12*), and *YAP1* genes were significantly higher in the GDM group than in controls, and another six CpGs sites within the *HAPLN3*, the interferon gamma receptor 2 (*IFNGR2*), *YAP1*, nuclear factor of activated T cells 4 (*NFATC4*), and *DNAJB6* genes were significantly lower. The DNAm level was obtained by MethylTarget sequencing and validation was performed using bisulfite conversion. Interestingly, receiver operating characteristics (ROC curves) were used to estimate the predictive efficacy of the methylation level of individual CpG site for the occurrence of GDM. They reported that the area under the curve (AUC) of the ROC curve for each methylation level of the significant CpG sites ranged from 0.593 to 0.650, and after adjusting for confounding factors, four CpGs (*HAPLN3*, *RDH12*, *DNAJB6*, *NFATC4*) sites showed independent effects on GDM. *HAPLN3* may function in hyaluronic acid binding and cell adhesion, and among its related pathways are the Integrin Pathway and ERK Signaling, while *RDH12* is a retinal reductase dependent on NADPH that influences the metabolism of short-chain aldehydes. *DNAJB6* is a DNAJ protein that serves as one of the two major classes of molecular chaperones promoting various cellular events, including protein folding and the assembly of oligomeric protein complex. *NFATC4* is part of a DNA-binding transcription complex that is activated by the calmodulin-dependent phosphatase calcineurin, and participates in the inducible expression of cytokine genes in T cells, especially that of interleukin-2 and interleukin-4 [49].

It is worth mentioning that women with a history of GDM are at greater risk of developing T2DM after pregnancy. With this in mind, Ballesteros et al. performed a longitudinal study and assessed DNAm using the Infinium Human Methylation 450 BeadChip in blood samples of GDM women between 26 and 30 weeks of pregnancy to test its predictive value for glucose metabolism alterations at 4 years postpartum. The differential methylated regions identified were sequenced on bisulfite-converted genomic DNA for replication purposes. The authors identified two CpGs sites related to long Intergenic Non-Protein Coding RNA 917 (*LINC00917*) and trafficking Protein Particle Complex Subunit 9 (*TRAPPC9*) associated with an abnormal glucose tolerance status at postpartum. Furthermore, they performed ROC curve analysis to assess the predictive value of *LINC00917*. The *LINC00917* cut-off point for predicting any kind of glucose disturbance was 93.83%, with a sensitivity of 70.6 and specificity of 73.0. The AUC with factors including pre-pregnancy BMI, age, fasting plasma glucose, gestational age, and family history of diabetes during pregnancy was 0.760 (95% CI 0.555–0.898), and after introducing *LINC00917* into the model, the AUC increased to 0.802 (95% CI 0.660–0.944) [53]. *LINC00917* is a long intergenic noncoding RNA, identified as contributing to pathogenesis of intervertebral disc degeneration and associated with Alzheimer’s disease [54,55]. *LINC00917* also has been associated with obesity in children [56]. *TRAPPC9* activates NF-kappa-B via increased phosphorylation of the IKK complex. It has been suggested that it promotes neuronal cell differentiation and may also serve in vesicular transport from the endoplasmic reticulum to the Golgi apparatus [53].

In addition, Linares-Pineda et al. suggested a shared DNAm mark between GDM and T2DM, some of which may at least partially explain the molecular mechanisms that mediate the increased risk of developing T2DM after GDM. The authors measured DNAm in peripheral blood leukocytes in two pregnancy cohorts, constructed a methylation risk score (MRS) based on CpGs associated with incident T2DM from a published epigenome-wide association study, and found that MRS for T2DM was associated with GDM [57].

Thus, the above-mentioned studies indicate that DNAm may contribute to predicting both GDM and glucose metabolism alterations postpartum. This knowledge may allow for early interventions among high-risk women identified by their DNAm profile, facilitating a personalized treatment of GDM and T2DM.

### 3.2. DNAm and Fetal Growth

Fetal growth is modulated by the fetal genome and by the developmental environment. The offspring of women with GDM are at increased risk of displaying altered growth patterns with increased neonatal body fat, higher weight at birth and increased ponderal index. GDM is characterized by hyperglycemia, leading to higher transplacental glucose transfer. Increased fetal glucose stimulates fetal insulin production, leading to hyperinsulinemia, which increases fetal adipose tissue and fetal overgrowth. GDM is also associated with increased triglycerides and free fatty acids, which are other maternal factors promoting fetal growth [12,13].

Other proposed factors influencing fetal growth and development in GDM are growth factors such as human placental growth hormone, maternal insulin-like growth hormones (IGF-I, IGF-II), and IGF-binding proteins (IGFBPs), which stimulate gluconeogenesis and lipolysis, increasing nutrient availability. In addition, maternal adipokines, including leptin, adiponectin, and tumor necrosis factor-α, have also been related to enhanced fetal growth through their contribution to insulin resistance, resulting in hyperglycemia [11].

DNAm may play a part in GDM-related developmental programming. To date, more than 15 studies have evaluated the relationship between DNAm and birth weight in GDM women. Different approaches have been used to evaluate this relationship. Placenta has been the focus of numerous studies. However, CB, maternal AT, and MB have also been evaluated (Table 1). Several studies applied genome-wide approaches, other studies measured global DNAm, and in most cases, research was focused on specific DNAm in key genes that regulate body weight, brown adipose tissue genesis and activation, developmental processes, lipid metabolism and transport, energy metabolism, insulin signaling, and facilitation of nutrient exchange [27,28,29,30,31,32,33,34,35,36,37,38,39,40,41,42,43,44,45].

#### 3.2.1. DNAm in the Placenta

Fetal growth is closely intertwined with the growth and development of the placenta. Placenta mediates the transfer of gases, nutrients, and waste and provides an immunological barrier between the fetus and the mother. Furthermore, the placenta is a source of a large number of adipokines that regulate insulin action and insulin resistance. In GDM pregnancy, placental weight is higher compared with normal pregnancy [58].

Previous studies have demonstrated that DNAm in the placenta plays an important role in fetal growth. Ruchat et al. found that 326 methylated genes in the placenta were associated with newborn weight [27]. Nomura et al. reported a suggestive negative association between global placental methylation and infant length and head circumference (which lost significance after correcting for multiple testing) [28]. Reichetzeder et al. demonstrated increased DNAm in the placenta of infants born large for gestational age compared to those that were small for gestational age and appropriate for gestational age [33]. Similarly, Gagné et al. detected a positive correlation between lipoprotein lipase (*LPL*) DNAm within the proximal promoter locus and birth weight. It is important to emphasize that this was a prospective study in which *LPL* placental DNAm was also positively associated with offspring body composition at 5 years old [35]. On the other hand, Steyn et al. found a positive correlation between the methylation of selected promotor CpG islands of the *IGFBP-1*, *IGFBP-2*, and *IGFBP-6* genes in the placentas of female babies and birth weight [37]. Zhao et al. reported a positive correlation between Delta-like 1 (*DLK1*) DNAm in the promoter region and birth weight [38]. Chen et al. also found that maternally expressed gene 3 (*MEG3*) DNAm within the differentially methylated region (DMR) on the maternal side of the placenta positively correlated with birth weight. Interestingly, this relationship was not found on the other side of the placenta [40]. Song et al. demonstrated a positive correlation between solute carrier family 6 member 4 (*SLC6A4*) DNAm within the promoter region and birth weight [42]. Notably, Wang et al. found that placental DNAm in Protocadherin Beta-15 (*PCDHB15*), Dickkopf WNT Signaling Pathway Inhibitor 2 (*DKK2*), ETS Transcription Factor ERG (*ERG*), Cell Adhesion Molecule 2 (*CADM2*), Cytochrome P450 Family 2 Subfamily D Member 7 (*CYP2D7P1*), Signal Regulatory Protein Beta 1 (*SIRPB1*), and Potassium Voltage-Gated Channel Subfamily A Regulatory Beta Subunit 2 (*KCNAB2*) were positively correlated with birth weight, and DNAm in Rap Guanine Nucleotide Exchange Factor 5 (*RAPGEF5*), Calcium Voltage-Gated Channel Auxiliary Subunit Alpha2delta 4 (*CACNA2D4*), Proprotein Convertase Subtilisin/Kexin Type 9 (*PCSK9*), and T-SNARE Domain Containing 1 (*TSNARE1*) were negatively correlated with birth weight. Of note, the results obtained by Wang et al. were adjusted for placental cell heterogeneity and were validated. Methylation level differences showed the same pattern between GDM and controls. However, the differences were not statistically significant [43].

In contrast to these studies, Lesseur et al. did not find an association between leptin (*LEP*) DNAm of the promoter region and birth weight [29]. Côté et al. did not find an association between DNAm levels at the PR/SET Domain 16 (*PRDM16*), Bone Morphogenetic Protein 7 (*BMP7*), C-Terminal Binding Protein 2 (*CTBP2*), and PPARG Coactivator 1 (*PPARGC1*) gene loci and birth weight [32]. Blazevic et al. did not demonstrate correlations between DNAm at CpG sites within the *SLC6A4* distal promoter region and birth weight [34]. In addition, Horvatiček et al. could not find an association between serotonin receptor 2A (*HTR2A*) promoter DNAm and birth weight [44].

It is noteworthy that only a few studies determined the functional relevance of DNAm for the regulation of gene expression (mRNA). A negative correlation was found between *SLC6A4*, *LPL*, glucose-6-phosphate dehydrogenase (*G6PD*), *IGFBPs*, *DLK1*, and *MEG3* DNAm and placental mRNA level [34,35,37,38,40]. Only one study found a positive correlation between *SLC6A4* DNAm and mRNA, and one study did not find an association between DNAm and fetal growth CB biomarker levels such as insulin, IGF-1, IGF-2, leptin, and adiponectin [42,43].

SLC6A4 is a membrane protein that regulates 5-hydroxyptamine (5HT or serotonin) homeostasis, mediating the uptake of serotonin into cells [59]. 5HT is a neurotransmitter that regulates the development of the serotonergic system and has a role in the etiopathogenesis of mental and metabolic conditions such as depression, autism, and obesity [60,61]. SLC6A4 is abundant in the brain and in the placenta, where it provides the 5HT needed for prenatal brain development [62]. Offspring of GDM mothers have an increased risk of lower cognitive development, attention deficit hyperactivity disorder, and autism spectrum disorder [63,64,65]. Blazevic et al. reported that *SLC6A4* methylation correlated negatively with maternal glucose concentrations in the 24th to 28th week of gestation in GDM women and demonstrated that placental *SLC6A4* methylation correlated inversely with *SLC6A4* mRNA levels [34]. In contrast, Song et al. found *SLC6A4* methylation levels were positively correlated with *SLC6A4* mRNA levels. This suggests that the serotonergic system in the placenta is probably subject to additional factors [42].

LPL contributes to metabolic homeostasis through triglycerides hydrolysis. This releases free fatty acids (FFAs) into the circulation [66]. LPL located in the placenta transfers FFAs to the fetus to support fetal growth and development [67]. GDM has been associated with an impairment of placental LPL, impacting fetal fat mass accretion, growth, and development [68]. Gagné et al. found a negative correlation between *LPL* DNAm and mRNA levels in the placentas of women with GDM [35].

G6PD is a cytoplasmic enzyme whose function is to catalyze the rate-limiting step in the oxidative branch of the pentose phosphate pathway (PPP), generating precursors for the synthesis of coenzymes, nucleotides, RNA, DNA, and NADPH [68]. One of the major functions of NADPH is to prevent oxidative stress [69]. Steyn et al. reported a negative correlation between maternal blood glucose levels and *G6PD* expression and a positive one with the methylation in both the placenta and the maternal white blood cells [37]. This suggests that the low expression of both maternal and placental *G6PD* contributes to oxidative stress, enhancing the progression of GDM. It is known that placental oxidative stress is involved in GDM etiology [70].

IGFBPs regulate the bioavailability of IGF-1 and IGF-2, which play a vital role in fetal growth regulation [71]. Reduced levels of IGFBPs result in an increase in free, unbound IGFs, allowing them to bind to their respective receptors. Steyn et al. found reductions of mRNA expression and increases in promoter methylation for *IGFBPs* in GDM-exposed placenta. Furthermore, maternal and placental *IGFBP-1* and *IGFBP-2* mRNA levels correlated negatively with maternal glucose levels [37]. This suggests that high maternal glucose levels during pregnancy may affect the bioavailability of IGFs via the attenuation of both maternal and placental *IGFBP* expression, possibly increasing the somatic growth of the fetus.

DLK1, which is also called a fetal antigen or preadipocyte factor 1, is a maternal imprinted gene encoding a transmembrane protein that is considered a growth factor [72]. Expressed in the long arm of chromosome 14 in humans, its defects associate with obesity, insulin resistance, and impaired glucose tolerance [73]. Zhao et al. reported that *DLK1* expression in both sides of the placenta decreased significantly in the GDM group vs. the control group, caused by the hypermethylation of the *DLK1* promoter region. In addition, the methylation of the *DLK1* gene on the maternal side of the placenta correlates highly with maternal 2 h OGTT glucose levels [38].

MEG3 is an imprinted gene located on human chromosome 14q32.3 within the DLK1- MEG3 locus [74]. One genome-imprinting study has demonstrated that the Dlk1-Dio3 locus is responsible for fetal growth in mice [75]. It would be logical to assume that the *MEG3* DNAm profile plays a potential role in GDM-related developmental programming [76]. Chen et al. found that mean *MEG3* DNAm levels showed a positive correlation with maternal fasting glucose concentrations [40].

#### 3.2.2. DNAm in CB

Various studies have demonstrated that the DNAm patterns in CB are associated with both exposure to the intrauterine environment and disease status. A number of studies have examined associations between CB DNAm and birth weight in GDM women. Ruchat et al. found that 117 methylated genes in CB were associated with newborn weight [27]. Allard et al. performed an epigenetic Mendelian randomization approach study and reported that lower DNAm at *LEP* locus was associated with higher CB leptin levels and birth weight. However, they did not find associated DNAm at the *LEP* locus and skin folds [30]. Su et al. described that an increased methylation of sites of H19 imprinted maternally expressed transcript (*H19*) DMRs and that decreased methylation at sites of *IGF-2* DMRs was closely related to birth weight [31]. Ott et al. observed that DNAm at region R2 and R3 of the adiponectin (*ADIPOQ*) gene locus is significantly altered in CB offspring, and that methylation was associated with birth weight [36]. Yan et al. described two specific CpG sites (cg12604331, cg08480098) in the gene body of Rho Guanine Nucleotide Exchange Factor 11 (*ARHGEF11*) that correlated negatively with maternal glucose concentrations and offspring birth weight. Interestingly, they performed a DNAm validation and found a significant reduction in *ARHGEF11* methylation in CB samples from women with GDM compared to those from the normal glucose-tolerant (NGT) women. Moreover, they noted a downregulation of *ARHGEF11* gene expression when neonatal birth weight was ≥4000 g, regardless of GDM status [41]. Finally, Mansell et al. showed a negative relationship between *LEP* promotor methylation and birth weight [39]. However, the association of DNAm in CB with birth weight remains controversial. A study by Nomura et al. could not observe an association between global methylation in CB and birth weight [28].

Importantly, only three studies determined the functional role of DNAm for the regulation of gene expression in CB. Su et al. observed an increased expression of *IGF2* and decreased expression of *H19* in the CB of fetuses of GDM compared to NGT women. Furthermore, they reported a reduced methylation level of *IGF2* and an increased methylation level of *H19* in the GDM group vs. the NGT group [31]. These results suggest that the modified genomic DNAm status of *IGF2* and *H19* may account for the change in their gene expression. *H19* and *IGF2* are imprinted genes, coregulated within the same locus at human chromosome 11p15 and associated with fetal birth weight [77,78].

Of interest, Ott et al. observed that *ADIPOQ* DNAm in CB was not associated with its gene expression. However, methylation was positively associated with CB adiponectin. Adiponectin was not significantly altered in GDM and was not correlated with newborns’ anthropometry [36]. It is probable that this adipokine, with functions in enhancing insulin sensitivity, glucose/lipid disposal/oxidation, and anti-inflammatory and anti-atherosclerotic mechanisms, has no implication in fetal growth [79].

Finally, Yan et al. studied *ARHGEF11*, a gene involved in early adipose tissue development which influences the susceptibility to obesity and T2DM [80]. They found that in macrosomic neonates exposed to GDM, *ARHGEF11* was significantly hypomethylated in CB. Additionally, *ARHGEF11* gene expression was downregulated in CB when neonatal birth weight was ≥4000 g, regardless of GDM exposure [41]. These results therefore suggest that DNAm at *ARHGEF11* does not have a functional impact on gene expression.

#### 3.2.3. DNAm in AT

Maternal obesity may impact fetal growth through the production of adipokines and inflammatory markers by adipose tissue [81]. Several studies have reported that maternal adiponectin is associated with birth weight [82,83]. Regarding DNAm, Ott et al. discovered that although fat tissue *ADIPOQ* DNAm of regions R2 and R3 was slightly altered in patients with GDM, subcutaneous adipose tissue (SAT) and visceral adipose tissue (VAT) DNAm was not associated with birth weight. However, methylation was inversely associated with *ADIPOQ* gene expression in SAT and VAT [36].

#### 3.2.4. DNAm in Maternal Blood

Circulating maternal white blood cells allow for easy access for experimental and clinical purposes. The methylation profiles of genes involved in fetal development in blood samples from pregnant women with GDM have been studied. Steyn et al. did not observe a correlation between the methylation of *IGFBPs* in maternal blood at 29–33 weeks’ gestation and birth weight. Furthermore, they could not report a significant association between the level of promoter region methylation and the magnitude of gene expression for *IGFBP-1*, *IGFBP-2*, *IGFBP-6*, and transketolase (*TKT*) in MB. However, *G6PD* mRNA expression showed a significant association with promoter region methylation in MB [37].

On the other hand, a specific methylation pattern from cell-free DNA (cfDNA) extracted from maternal plasma has been evaluated. The unmethylated/methylated ratio of *insulin* and *amylin* genes is a noninvasive marker of β-cell death. A lower ratio indicates a higher rate of β-cell destruction [84,85]. Linares et al. recently showed that GDM women showed a higher β-cell death of both markers than subjects without diabetes. Furthermore, they found that the insulin methylation index in cfDNA was associated with insulin resistance and with newborn birth weight [45]. These results suggest that β-cell death influences the development of GDM and is associated with newborn weight. GDM is characterized by the impaired function of β-cells, which may undergo death due to a higher turn-over process to compensate for physiological insulin resistance during pregnancy [86].

## 4. Conclusions and Future Perspectives

It has been suggested that a peripheral blood DNAm profile in early pregnancy could be considered a diagnostic and prognostic marker of GDM [51,52,53]. In addition, GDM has been suggested to promote global and gene-specific DNAm changes in the placenta, CB, maternal AT, and maternal blood which may contribute to fetal growth and development. Importantly, investigating early adipose tissue development in humans is challenging since the tissue of interest cannot be directly assessed for clinical and ethical reasons. As a proxy for overall fetal adipose tissue development status, all studies assessed birth weight, even though a number of studies analyzed other markers for adiposity in offspring, such as fetus body length, head circumference, sum of four skin folds, chest circumference, triceps and subscapular skinfold thickness, and ponderal index at birth [28,30,31,32,33,39,42].

To date, more than 15 studies have evaluated the relationship between DNAm and birth weight in GDM women. Most of these studies analyzed the placenta; other researchers studied CB and MB, and only one study analyzed maternal AT [27,28,29,30,31,32,33,34,35,36,37,38,39,40,41,42,43,44,45]. Patterns of DNAm can occur in a tissue-specific manner or can be similar in various tissues. Ruchat et al. reported that over 25% of differentially methylated genes in GDM were common to both the placenta and CB [27]. However, Ott et al. did not observe a correlation between the methylation of VAT and maternal blood cells, but a similar methylation pattern occurred in CB and MB [36]. Nomura et al. noted a lack of correlation between the levels of global methylation in CB and the placenta, and Steyn et al. found different methylation data for the placenta and MB [28,37]. This is particularly important given the increased evidence supporting the adjustment of DNAm for the cell proportion of different samples [52].

Specifically, several authors reported a relationship between DNAm and birth weight. However, other studies showed inconsistent results. There are several reasons for these discrepancies. These include different sample sizes, heterogeneity in the diagnosis criteria of GDM, the variety of methodological processes for DNAm profiles, variations in the cellularity of tissues, the different CpG site locations analyzed, and adjusting for potential confounding factors such as infant sex, gestational age, maternal age, body mass index at the beginning of pregnancy, gestational weight gain, GDM treatments, ethnicity, familiar diabetes predisposition, parity, mode of delivery, and smoking status before pregnancy.

Interestingly, most of the studies have several limitations. The main ones are small sample sizes and the absence of a validation group, so the results might not be applicable to other populations. Also, because of the cross-sectional study design of all studies conducted at the end of pregnancy, the causality of DNAm on fetal growth cannot be inferred. Hence, this epigenetic modification should be tested in MB in early pregnancy in order to be considered a potential predictor of fetal growth. Additionally, numerous studies did not investigate the functional impact of DNAm on gene expression and, importantly, they did not adjust for genotype predictors of DNAm and gene expression. Regarding validation, only two studies analyzed it. The first of these was conducted by Yan et al. and the other study was conducted by Wang et al. [41,43]. They found the same pattern in the methylation level differences between GDM and control patients with validation. However, Wang et al. observed that differences were not statistically significant [43]. Regarding gene expression, only eight studies measured mRNA levels. Most of these studies reported a negative correlation between the DNAm and mRNA level of genes thought to be involved in the metabolic disease pathway with consequences on fetal growth and development, such as *SLC6A4*, *LPL*, *G6PD*, *IGFBPs*, *DLK1*, *MEG3*, *IGF2*, and *H19* [31,34,35,37,38,40]. Regarding the study of maternal genetic variants that regulate DNAm, only four studies included it. Lesseur et al., Mansell et al., and Horvatiček et al. described that genetic variation strongly influenced *LEP* and *HTR2A* DNAm [29,39,44]. On the contrary, Blazevic et al. did not find an influence of polymorphisms in the *SLC6A4* promoter, analyzed either separately or in combination, on *SLC6A4* DNAm [34].

In addition, there are various methods to examine DNAm profiles, such as liquid chromatography (LC) combined with fluorescence, ultraviolet, and mass spectrometry (MS), bisulfite conversion-based and endonuclease digestion-based methods, array-based platforms, and whole-genome sequencing. Although each method has advantages and limitations, the whole-genome sequencing approach seems to offer important advantages, given that besides detecting DNAm in gene promoters, it is able to detect DNAm in intergenic regions that might have regulatory functions [87]. It must be noted that no study to date has performed whole-genome sequencing to identify DNAm signatures related to birth weight. On the other hand, the most widely used and affordable methods to profile epigenome-wide DNAm are arrays. Four studies included in this review used methylation arrays. Three studies used the Illumina Infinium HumanMethylation450 (450K) BeadChip array, which covers >480,000 CpGs, and one used the Infinium methylationEPIC array, covering >850,000 CpGs [27,41,43]. The Infinium 450 K BeadChips array only covers about ~1.5% of all genomic CpGs, and the Infinium methylationEPIC array covers 30% of the human methylome [88,89]. Most of the studies reviewed used locus-specific bisulfite sequencing, which is suitable for the detection of representative high-density DNAm regions. However, it could not cover all CpGs in the genome [90]. One reviewed study used LC combined with MS, which has an adequate sensitivity but is unable to offer detailed information regarding methylation at specific sites [90].

DNAm, besides influencing early fetal body composition, may regulate the later risk of obesity [91,92]. Gagné et al. demonstrated that alterations in fetal placental DNAm levels at the *LPL* gene locus are associated with anthropometric profiles in children at 5 years of age [35]. On the other hand, Mansell et al. found that birth adiposity was associated with 12-month *LEP* methylation. However, *LEP* methylation was not associated with 12-month anthropometric measures, possibly indicating of the existence of various other potential influences on *LEP* methylation in the postnatal environment [39].

Intriguingly, specific environmental factors, such as diet, stress, and physical activity, may influence DNAm [93,94,95]. Furthermore, folate and choline supplementation preparations during pregnancy influence DNAm-modifying gene expressions [96]. We noted that no study included in this review had data on these factors. Further studies are needed to indicate whether these factors affect the associations between gene methylations and birth weight.

In summary, altered DNAm in a number of placental, CB, and MB genes was associated with birth weight in the offspring of GDM women, and some of these gene methylations were correlated to gene expression. These genes are involved in energy and glucose metabolism, in fetal and placental growth, and in neurobiological and behavioral functions, possibly affecting fetal growth and development. Thus, the reviewed studies suggest that DNAm may serve as a biomarker of macrosomia, which is high amongst women with GDM, and for which there is currently no reliable prediction method. However, future studies with larger samples, with extensive coverage in the methylome and the transcriptome, with a follow-up of women throughout pregnancy, taking into account potential confounding factors, and with a more detailed analysis of DNAm biological function will be needed to determine the true value of DNAm with respect to fetal growth. Moreover, as the prevalence of GDM continues to rise, more studies are urgently needed in order to assess the possibility of preventing epigenetic programming at birth with interventions during pregnancy. This might promote children without future obesity and with a healthier metabolic trajectory.

## Figures and Tables

**Table 1:** Studies assessing DNAm and fetal growth in gestational diabetes.

Author, Year [Reference]	Case/Control	Biological Source	Approach to Identify DNAm	Method	Main Finding
Ruchat, 2013 [27]	30/14	Placenta and CB	Genome-wide approach	Infinium Human Methylation 450 Beadchips	A total of 3271 and 3758 genes in the placenta and CB were differentially methylated between those exposed and those not exposed to GDM. A total of 326 methylated genes in the placenta and 117 in CB were associated with newborn weight.
Nomura, 2014 [28]	7/21	Placenta and CB	Global	Luminometric Methylation Assay	GDM was associated with placental hypomethylation. Suggestive negative associations were found between placental global methylation and infant body length and head circumference.
Lesseur, 2014 [29]	47/432	Placenta	*LEP* promoter region	Quantitative bisulfite pyrosequencing	Placentas from infants exposed to GDM had higher DNAm compared to those from the non-GDM group. *LEP* methylation was not associated with infant birth weight.
Allard, 2015 [30]	173	CB	*LEP* genomic region	Human Methylation 450 BeadChips at *LEP* locus	Increased maternal glycemia associated with lower methylation levels at *LEP* locus. Lower DNAm at *LEP* locus associated with higher leptin levels in CB and higher birth weight.
Su, 2016 [31]	55/60	CB	*IGF-2* and *H19*	Sequenom massARRAY	Decreased methylation of *IGF-2* and increased methylation of *H19* in the GDM group compared to NGT group. Increased methylation of *H19* and decreased methylation of *IGF-2* were closely related to birth weight. An increase in *IGF2* and decrease in *H19* expression in the placenta and CB were observed in the GDM group compared to the NGT group.
Côté, 2016 [32]	33/100 in silico: 172	Fetal placenta	*PRDM16*, *BMP7*, *CTBP2*, *PPARGC1*	Bisulfite pyrosequencing, array, and an in silico replication study using data from HumanMethylation450 BeadChip Arrays	*BMP7* and *PRDM16* DNAm levels were lower and *PPARGC1α* was higher in GDM-exposed placentas vs. those not exposed. DNAm was not associated with birth weight. Leptin CB levels correlated with *PRDM16* and *PPARGC1* DNAm levels. Higher CB leptin levels were correlated with higher birth weight and chest circumference.
Reichetzeder, 2016 [33]	56/974	Placenta	Global	LC-MS/MS	Mothers with GDM displayed a significantly increased global placental DNAm. LGA was associated with increased placental DNAm compared to SGA and AGA.
Blazevic, 2017 [34]	18/32	Fetal side of the placenta	*SLC6A4*	Direct bisulfite sequencing	DNAm was lower in the GDM as compared to the NGT group and showed a negative correlation with maternal plasma glucose levels. Placental *SLC6A4* mRNA levels were inversely correlated with average DNAm. *SLC6A4* methylation levels did not correlate with birth weight; however, placental *SLC6A4* mRNA levels showed a negative correlation with infant birth weight.
Gagné, 2017 [35]	24/42	Fetal side of the placenta	*LPL*	Bisulfite pyrosequencing	DNAm levels were lower when the placenta had been exposed to GDM, compared with those with no GDM exposure. *LPL* DNAm was inversely correlated with *LPL* mRNA levels. *LPL* DNAm levels were positively correlated with birth weight.
Ott, 2018 [36]	25/30	Maternal SAT, VAT, and CB	*ADIPOQ*	Pyrosequencing	Fat tissue DNAm was slightly altered in patients with GDM. Methylation was inversely associated with *ADIPOQ* gene expression in SAT and VAT. *ADIPOQ* DNAm was significantly altered in offspring CB, and methylation was associated with birth weight.
Steyn, 2019 [37]	6/ 6	Placenta and maternal blood	*G6PD*, *TKT*, *IGFBP-1*, *IGFBP-2*, *IGFBP-6*	EpiTect Methyl II PCR assay	Decreased mRNA expression and increased promoter methylation were noted for *G6PD* in GDM women and for genes encoding *IGF-BPs* proteins in GDM-exposed placentas. MB methylation of *IGFBPs* did not correlate with birth weight. However, placental methylation of *IGFBP-1*, *IGFBP-2*, and *IGFBP-6* correlated positively with birth weight.
Zhao, 2019 [38]	15/15	Maternal and fetal sides of the placenta	*DLK1*	MethylTargetTM	Hypermethylation of *DLK1* promoter region caused decreased *DLK1* expression in both maternal and fetal sides of the placenta in the GDM group compared with the control group. The DNAm of *DLK1* in the fetal side of the placenta was closely related to fetal birth weight.
Mansell, 2019 [39]	35/?	CB	*LEP*	Bisulphite conversion using the MagPrep Lightning Conversion Kit	There was some evidence of a relationship between GDM and *LEP* methylation, with a negative relationship between CpG7 *LEP* methylation and birth weight.
Chen, 2021 [40]	23/23	Maternal and fetal sides of the placenta	*MEG3*	MethylTargetTM	DNAm in *MEG3* was higher in the maternal side of the placenta in the GDM vs. control group, while the mRNA expression of *MEG3* was significantly reduced. Placental DNAm showed a positive correlation with both maternal fasting glucose concentrations and offspring birth weight.
Yan, 2021 [41]	6/6	CB	Global Whole genome	Infinitum Human Methylation 450 BeadChip array and DNAm validation using SEQUENOM MassARRAY	A total of 1251 genes were methylated differently in GDM subjects vs. controls. Macrosomic GDM infants showed hypomethylated *ARHGEF11*. *ARHGEF11* gene expression was downregulated when neonatal birth weight was ≥4000 g, regardless of GDM. Altered DNAm levels of *ARHGEF11* showed negative correlation with both glucose concentrations and neonatal birth weight.
Song, 2022 [42]	30/60	Fetal side of the placenta	*SLC6A4*, *HTR2A*	Pyrosequencing	The average DNAm of *SLC6A4* was higher in the GDM group than in the control group, while the DNAm of *HTR2A* showed no difference. *SLC6A4* methylation correlated positively with placental *SLC6A4* mRNA levels. *SLC6A4* methylation demonstrated a positive correlation with maternal plasma glucose level and neonatal birth weight percentile but correlated negatively with neonatal head circumference percentile.
Wang, 2022 [43]	30/30	Fetal side of the placenta	Global	Infinium MethylationEPIC Beadchip and Bisulfite-pyrosequencing validation	A total of 256 DMPs (130 hypermethylated and 126 hypomethylated) were reported between the GDM and control groups. Methylations in *PCDHB15*, *DKK2*, *ERG*, *CADM2*, *CYP2D7P1*, *SIRPB1*, and *KCNAB2* positively correlated with birth weight, while methylations in *RAPGEF5*, *CACNA2D4*, *PCSK9*, and *TSNARE1* showed a negative correlation. No gene methylation correlated with metabolic biomarkers (fetal growth factors, leptin, and adiponectin) in CB after correcting for multiple tests.
Horvatiček, 2022 [44]	80/119	Fetal side of the placenta	*HTR2A*	Bisulfite pyrosequencing quantification	GDM was associated with reduced *HTR2A* DNAm in female but not male placentas. Birth weight was not a significant predictor of methylation in either female or male placentas.
Linares, 2023 [45]	25/25	Maternal plasma	*Insulin*, *amylin*	qPCR	The demethylation indexes of *insulin* and *amylin* were decreased. The *insulin* methylation index was associated with insulin resistance and with newborn birth weight.

DNAm: DNA methylation, CB: cord blood, GDM: gestational diabetes mellitus, *LEP*: leptin, *IGF-2*: insulin-like growth factor 2, *H-19*: H19 Imprinted Maternally Expressed Transcript, NGT: normal glucose tolerant, *PRDM16*: PR/SET Domain 16, *BMP7*: Bone Morphogenetic Protein 7, *CTBP2*: C-Terminal Binding Protein 2, *PPARGC1*: PPARG Coactivator 1, LC-MS/MS: liquid chromatography with tandem mass spectrometry, LGA: large for gestational age, AGA: appropriate for gestational age, SGA: small for gestational age, *SLC6A4*: solute carrier family 6 member 4, *LPL*: lipoprotein lipase, SAT: subcutaneous adipose tissue, VAT: visceral adipose tissue, *ADIPOQ*: adiponectin, *ARHGEF11*: Rho Guanine Nucleotide Exchange Factor 11, *G6PD*: glucose-6-phosphate dehydrogenase, *TKT*: transketolase, *IGFBP*: insulin-like growth factor-binding protein, *DLK1*: Delta-like 1, *MEG3*: maternally expressed gene 3, *HTR2A*: serotonin receptor 2A, DMP: differential methylation position, *PCDHB15*: Protocadherin Beta-15, *DKK2*: Dickkopf WNT Signaling Pathway Inhibitor 2, *ERG*: ETS Transcription Factor ERG, *CADM2*: Cell Adhesion Molecule 2, *CYP2D7P1*: Cytochrome P450 Family 2 Subfamily D Member 7, *SIRPB1*: Signal Regulatory Protein Beta 1, *KCNAB2*: Potassium Voltage-Gated Channel Subfamily A Regulatory Beta Subunit 2, *RAPGEF5*: Rap Guanine Nucleotide Exchange Factor 5, *CACNA2D4*: Calcium Voltage-Gated Channel Auxiliary Subunit Alpha2delta 4, *PCSK9*: Proprotein Convertase Subtilisin/Kexin Type 9, *TSNARE1*: T-SNARE Domain Containing 1.

## Data Availability

Not applicable.
